# Effect of Different Heat Treatment Processes on the Microstructure and Properties of Cu-15Ni-3Al Alloys

**DOI:** 10.3390/ma18122678

**Published:** 2025-06-06

**Authors:** Jinchun Ren, Qiangsong Wang, Liyan Dong, Junru Gao, Xinlu Chai

**Affiliations:** 1 State Key Laboratory of Nonferrous Structural Materials, China GRINM Group Co., Ltd., Beijing 100088, China; rjc030800@163.com (J.R.); dly2568@163.com (L.D.); gjr825519@163.com (J.G.); 17836056387@163.com (X.C.); 2 GRIMAT Engineering Institute Co., Ltd., Beijing 101407, China; 3 General Research Institute for Nonferrous Metals, Beijing 100088, China

**Keywords:** Cu-Ni-Al alloy, spinodal decomposition, mechanical property, microstructure

## Abstract

This study systematically investigates the influence of different heat treatment processes on the microstructural evolution and mechanical properties of Cu-15Ni-3Al alloys, with particular emphasis on the synergistic strengthening mechanisms of spinodal decomposition and precipitation hardening. Two distinct aging routes—solution aging and direct aging—were designed to facilitate a comparative assessment of microstructural characteristics and their correlation with mechanical performance. Comprehensive characterization was conducted using scanning electron microscopy (SEM), X-ray diffraction (XRD), transmission electron microscopy (TEM), and room-temperature tensile testing to elucidate the structure–property relationships. The results reveal that direct aging promotes the formation of fine, coherent L1_2_-type Ni_3_Al precipitates and the evolution of Ni-enriched regions initially generated through spinodal decomposition into stable Ni_3_Al precipitates. These microstructural features act as effective barriers to dislocation motion, thereby significantly enhancing both strength and ductility. The findings provide valuable insights into optimizing heat treatment strategies to improve the performance of Cu-Ni-Al alloys.

## 1. Introduction

Advanced copper-based materials, serving as critical foundational materials with extensive applications, are integral to industries such as electronic information, aviation, aerospace, energy and power, transportation, pharmaceuticals, chemicals, and marine engineering, significantly impacting global technological competition and economic landscapes [1]. In the realm of wear-resistant copper alloys, conventional alloys like complex brass, tin bronze, lead bronze, aluminum bronze, and beryllium bronze have found widespread use in components such as bearing retainers, automotive synchronizer gear rings, and hydraulic wear-resistant parts. With the rapid growth of China’s aviation, aerospace, and machinery sectors, the demand for copper alloys with enhanced strength, wear resistance, corrosion resistance, and heat resistance—such as those used in aero-engines and high-speed bearings—has surged, along with stricter performance expectations [2]. Brass and lead bronze suffer from low strength; tin bronze exhibits performance variability due to tin segregation and compositional inconsistency; aluminum bronze offers superior hardness, strength, wear resistance, and corrosion resistance but becomes brittle at low temperatures, reducing its impact toughness; and beryllium bronze, despite its outstanding elasticity and strength, contains toxic beryllium, posing severe health hazards. To address these limitations, third elements like Al, Sn, Si, and Mn have been incorporated into corrosion-resistant Cu-Ni alloys [3]. Leveraging spinodal decomposition and precipitation strengthening mechanisms, Cu-Ni-based alloys such as Cu-Ni-Al, Cu-Ni-Sn, Cu-Ni-Si, and Cu-Ni-Mn are emerging as high-strength, elastic, and wear-resistant alternatives to traditional copper alloys, and are increasingly adopted for demanding operational environments.

Cu-Ni-Al alloys exhibit high strength, elasticity, and exceptional wear and corrosion resistance, owing to the formation of stable Ni-Al intermetallic compounds such as Ni_3_Al, NiAl, Ni_2_Al_3_, and NiAl_3_. Recent research on Cu-Ni-Al alloys includes Calum G.F [4] et al., who heat-treated Cu-(5-15-25)Ni-5Al at 973 K, noting discontinuous L1_2_-type precipitates at grain boundaries that extended into adjacent grains; Li [5] et al., who leveraged cubic Ni_3_Al precipitation strengthening to develop a Cu-Ni-Al alloy with a softening temperature of 1273 K, overcoming the heat resistance limitations of copper alloys; and Satoshi [6] et al., who explored microstructural, strength, and conductivity changes in Cu-20Ni-6.7Al during isothermal aging from 723 K to 1023 K, showing that coherent spherical Ni3Al precipitates maximized strength after 8 h at 873 K. Additional studies enhanced alloy strength by introducing a fourth element: Dong [7] et al. designed Cu-17Ni-3Al-Si-Ti-Zr and examined G-phase precipitation within grains and its correlation with grain boundary orientation; Shen [8] et al. aged solution-treated Cu-10Ni-3Al-0.8Si at varying temperatures, observing continuous Ni_3_Al and Ni_2_Si precipitation at 723K, with discontinuous precipitation at grain boundaries and suppressed Ni_3_Al formation at higher temperatures; and Zhu [9] et al. achieved a tensile strength of 860.3 MPa in a Cu-Ni-Al-Si alloy through solution treatment at 960 °C and aging at 753 K [10].

The Cu-Ni phase diagram indicates that Ni is fully miscible in Cu, forming a continuous solid solution. During heat treatment, this Ni-Cu binary alloy undergoes spinodal decomposition, breaking down into alternating micro-regions of rich and lean phases that retain the original solid solution’s crystal structure [11]. The fine wavelength of this decomposed structure yields effective dispersion strengthening, hindering dislocation motion and boosting alloy strength. The coherent rich and lean phases create a periodic elastic strain field that resists dislocation movement without excessive pile-up, preserving the alloy’s plasticity and toughness.

Drawing on these insights, this study investigates the impact of solution aging versus direct aging heat treatments on the microstructure and mechanical properties of Cu-Ni-Al spinodal decomposition alloys. By clarifying precipitation behavior and strengthening mechanisms, it aims to streamline heat treatment processes and establish a theoretical foundation for efficiently producing high-strength, high-elasticity, wear-resistant, and corrosion-resistant Cu-Ni-Al alloys.

## 2. Materials and Methods

**Figure 1:**
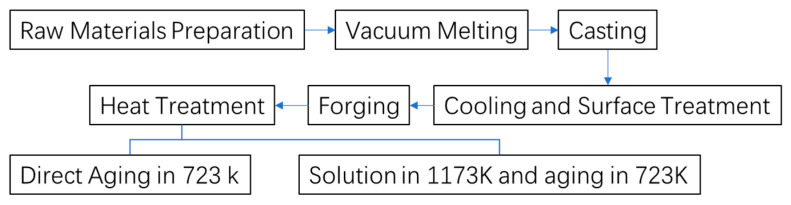
Cu-15Ni-3Al alloy preparation process.

In this study, Cu-15Ni-3Al alloy was fabricated by melting high-purity cathode electrolytic copper (99.95%), high-purity Ni (99.99%), and high-purity Al (99.99%). The detailed composition is provided in Table 1. Initially, 19.68 kg of electrolytic copper and 4.5 kg of high-purity nickel were placed into a ZG-0.025 vacuum induction furnace crucible. After evacuating to 10^−1^ Pa, the charge was heated to 1100 °C and held for 30 min. Subsequently, 0.9 kg of high-purity aluminum and the remaining 4.92 kg of electrolytic copper were added. The temperature was further increased to 1200 °C and maintained for another 30 min before vacuum casting was carried out to obtain a cylindrical ingot with a diameter of 150 mm and a height of 205 mm. The furnace was then allowed to cool naturally under vacuum conditions. To eliminate potential surface defects such as cracks or shrinkage cavities that may have formed during casting, and to ensure the structural integrity for subsequent forging, both ends of the ingot were removed, and surface machining was conducted. The processed ingot was then preheated at 850 °C for 3 h, followed by multi-directional forging using a “three upsetting–three drawing” strategy with alternating deformation directions. The deformation per pass was controlled to exceed 30%. The final forged billet had a diameter of 100 mm and a length of approximately 300 mm, providing a microstructurally homogeneous material for subsequent heat treatment. The preparation and heat treatment process of the Cu-15Ni-3Al alloy is summarized in Figure 1.

To investigate the heat treatment behavior of the Cu-15Ni-3Al alloy, two distinct heat treatment procedures were designed and implemented. The first route involved direct aging at 723 K for various durations: 2 h, 4 h, 6 h, …, up to 16 h. In the second route, the furnace was first heated to 1173 K. Once the target temperature was reached, the samples were placed into the furnace for solution treatment at 1173 K for 3 h, followed by water quenching to room temperature. The samples were then aged at 723 K for varying durations (2 h, 4 h, 6 h, …, up to 16 h). The heating rate during both solution treatment and aging was approximately 10 K/min, and water quenching was applied to ensure rapid cooling after solution treatment. The alloy’s heat treatment procedure is detailed in Table 2.

Room temperature tensile properties were evaluated using a universal tensile testing machine (WDW-300, Changchun Kexin Testing Machine Co., Ltd., Jilin, China) at a strain rate of 3 mm/min. To ensure data accuracy, three parallel specimens per alloy were tested, and their average values were used to determine the mechanical properties, including tensile strength (Rm), yield strength (Rp0.2), elongation after fracture (A), and reduction in area (Z). Alloy morphology was examined with a Sigma-300 scanning electron microscope (SEM, Zeiss, Oberkochen, Germany). The nature and structure of precipitated phases in the alloy were analyzed using a Tecnai G2 F20 transmission electron microscope (TEM, FEI, Hillsboro, OR, USA)), with high-resolution transmission electron microscopy (HRTEM) employed for detailed morphological characterization of these phases. TEM specimens were prepared through wire cutting, grinding, and punching, followed by twin-jet electropolishing. The electropolishing solution, composed of nitric acid and methanol in a 1:3 volume ratio, was operated at 15 V, with its temperature maintained at approximately −30 °C using liquid nitrogen. Lattice constants of the alloy were determined using an Xpert PRO MPO polycrystalline X-ray diffractometer (XRD, SmartLab, Rigaku Corporation, Akishima, Japan), equipped with a Cu target. The Cu-Kα radiation used had a geometric average wavelength of 0.15418 nm, accounting for both Cu-Kα_1_ (0.15406 nm) and Cu-Kα_2_ (0.15444 nm) components. A graphite monochromator was employed to suppress the Cu-Kα_2_ component, thereby improving peak resolution and eliminating spectral overlap, operating at an accelerating voltage of 40 kV, with a scanning range of 20° to 100° and a speed of 5°/min. Vickers hardness was measured with a 5 kg load applied to the alloy specimens for 15 s. Prior to testing, specimen surfaces were sequentially polished with 240#, 400#, 600#, 1000#, and 2000# sandpaper to eliminate surface oxides and other contaminants that could skew the hardness results, while also improving readability. At least five measurements were taken per specimen, and the average hardness value was calculated. The electrical conductivity of the samples was measured using a Sigma 2008A eddy current conductivity meter (Xiamen Tianyan Instruments Co., Ltd., Xiamen, China). All measurements were conducted at room temperature, and each data point represents the average of at least five repeated measurements to ensure accuracy and reproducibility.

## 3. Results

### 3.1. Variation in Properties

**Figure 2:**
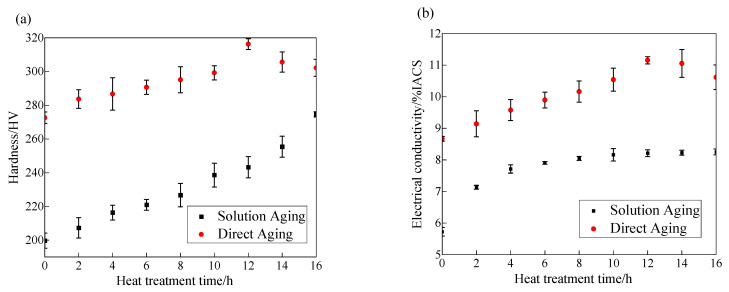
Properties of the alloy under different heat treatment conditions: (**a**) hardness; (**b**) conductivity.

The hardness of the material in various states was measured using a Vickers hardness tester. Figure 2a illustrates the hardness evolution of the Cu-15Ni-3Al alloy as a function of aging time under two conditions: direct aging at 723 K and solution treatment at 1173 K followed by aging at 723 K. For direct aging at 723 K, the hardness rises steadily from 272.64 HV in the as-forged state, peaking at 316.3 HV after 12 h. After solution treatment at 900 °C, the initial hardness is 199.75 HV, increasing with aging time to 274.64 HV after 16 h—comparable to the as-forged hardness but below the peak value, suggesting that longer aging is needed to reach maximum hardness. Figure 2b depicts the electrical conductivity changes of the Cu-15Ni-3Al alloy with aging time under the same heat treatment conditions. In both cases, conductivity increases with aging duration. For direct aging at 723 K, it climbs from 8.68%IACS in the as-forged state to a peak of 11.15%IACS after 12 h. Following solution treatment at 1173 K, the initial conductivity is 5.72%IACS, rising gradually to 8.25%IACS after 16 h. The continuous rise in conductivity with aging time is strongly tied to solute atom precipitation. In the solution-treated state, abundant Ni and Al atoms dissolved in the Cu matrix cause significant lattice distortion, intensifying electron scattering and minimizing conductivity. During aging, spinodal decomposition and ordering reactions prompt Ni and Al atoms to precipitate from the supersaturated Cu matrix, swiftly reducing electron scattering and boosting conductivity. While precipitate interfaces also scatter electrons, this effect is minor compared to the reduction caused by solute precipitation. After 12 h of aging, most solute atoms have precipitated, slowing the conductivity growth rate. Comparing the two treatments, the hardness and conductivity curves for direct aging consistently exceed those for solution treatment followed by aging, demonstrating superior properties with direct aging.

**Figure 3:**
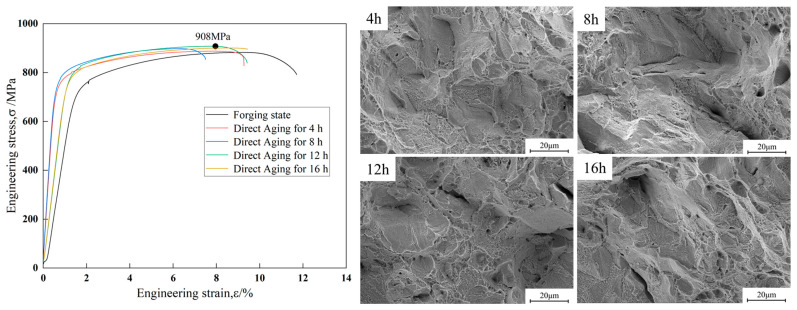
Engineering stress–strain curves and corresponding fracture morphologies of the alloy.

Figure 3 shows the engineering stress–strain curves and the corresponding fracture morphologies of the Cu-15Ni-3Al alloy in the forged condition and after direct aging at 723 K for 4 h, 8 h, 12 h, and 16 h. The strength and elongation of the Cu-15Ni-3Al alloy in different states is showed in Table 3. After aging treatment, the tensile strength of the alloy increases with aging time, rising from 872 MPa in the forged condition to a maximum of 910 MPa after 12 h of direct aging. However, the tensile strength decreases after aging for 16 h. The fracture morphologies reveal that under all conditions, the fracture surfaces are uniformly covered with a large number of dimples. The dimple sizes vary significantly, with a relatively large average size. Many small dimples are distributed around larger ones—characteristic features of ductile fracture in metals. According to the criteria for evaluating material plasticity, necking is a direct indicator of the degree of plastic deformation: the greater the necking, the better the plasticity. Dimple size is influenced by the material’s ability to undergo plastic deformation—the larger and deeper the average dimples, the better the plasticity of the material. Ripple-like features can also be observed on the walls of the elongated dimples. These are formed when the dimple surface is perpendicular to the direction of principal stress and high stress induces additional slip on the surface. This is a typical feature observed in metals undergoing ductile fracture.

### 3.2. Microstructural Evolution

#### 3.2.1. Observation of Metallographic Microstructure

**Figure 4:**
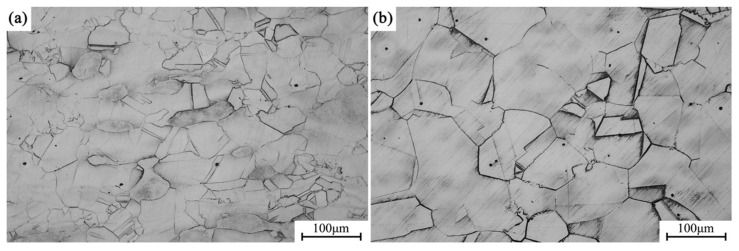
Alloy grain structures in different conditions: (**a**) forged; (**b**) solution treatment.

Figure 4a shows the metallographic microstructure of the alloy in its forged condition, while Figure 4b depicts the microstructure after solution treatment at 1173 K. With solution treatment, the grains exhibit substantial growth compared to the forged state, with the average grain size increasing from approximately 70 μm in the as-forged condition to about 150 μm in the solution-treated condition.

**Figure 5:**
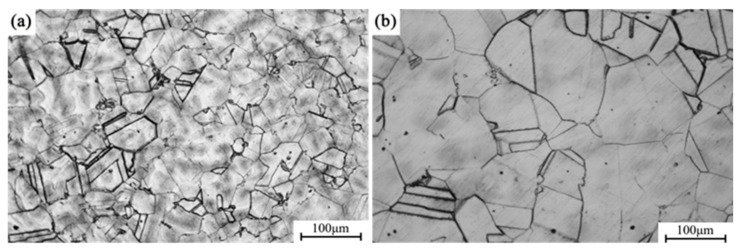
Alloy grain structures in different conditions: (**a**) direct aging for 12 h; (**b**) solution aging for 12 h.

Figure 5a presents the metallographic microstructure of the alloy after direct aging for 12 h, whereas Figure 5b shows the microstructure after undergoing solution treatment followed by aging for 12 h. The average grain size in the directly aged sample (a) is approximately 72 μm, whereas the grain size in the solution-aged sample (b) reaches 157 μm, indicating that grain growth is effectively suppressed during the direct aging process. Grain boundaries act as effective barriers to dislocation motion, resulting in grain boundary strengthening. The contribution of grain refinement to the yield strength (σ_g_) can be estimated using the Hall–Petch equation:

σ_g_ = k⋅d^−1/2^

where k is a material constant (for copper alloys, k = 0.18 MPa·m^−1/2^), and d is the average grain size. the estimated contribution of grain boundary strengthening is approximately 21.21 MPa for the directly aged sample and 14.37 MPa for the solution-aged sample.

#### 3.2.2. XRD Analysis

**Figure 6:**
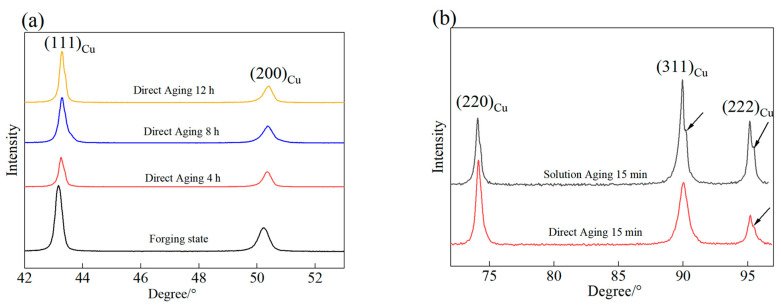
XRD patterns of the alloy in different states: (**a**) 42–52°; (**b**) 70–100°.

To determine the reasons for performance enhancement during the aging heat treatment of the alloy, XRD analysis was conducted to examine the changes in lattice constants of the Cu-15Ni-3Al alloy during the aging process. Figure 6 shows the XRD patterns of the Cu-15Ni-3Al alloy under different conditions. Figure 6a displays the X-ray diffraction spectra in the 2θ range of 42–52°. From the figure, broadening of the (111)Cu and (200)Cu matrix diffraction peaks can be observed. After aging, the diffraction peaks shift to the right to varying degrees, indicating an increase in the diffraction angles (2θ). The detailed diffraction angles and lattice parameter variations are showed in Table 4. This shift suggests that the lattice constant of the matrix decreases during aging. Since grain size changes little during aging and large-scale dislocation multiplication does not occur, the observed peak broadening is primarily attributed to the formation of precipitates. This is caused by Ni and Al solute atoms precipitating out from the matrix. Figure 6b presents the XRD results of the alloy after direct aging for 15 min and after solution treatment followed by 15 min of aging. It can be observed that sideband peaks appear on both sides of the (311)Cu and (222)Cu diffraction peaks after just 15 min of aging. The appearance of these sideband peaks is due to lattice strain modulation caused by compositional modulation from spinodal decomposition, resulting in alternating regions of solute enrichment and depletion in the matrix.

#### 3.2.3. SEM and TEM

**Figure 7:**
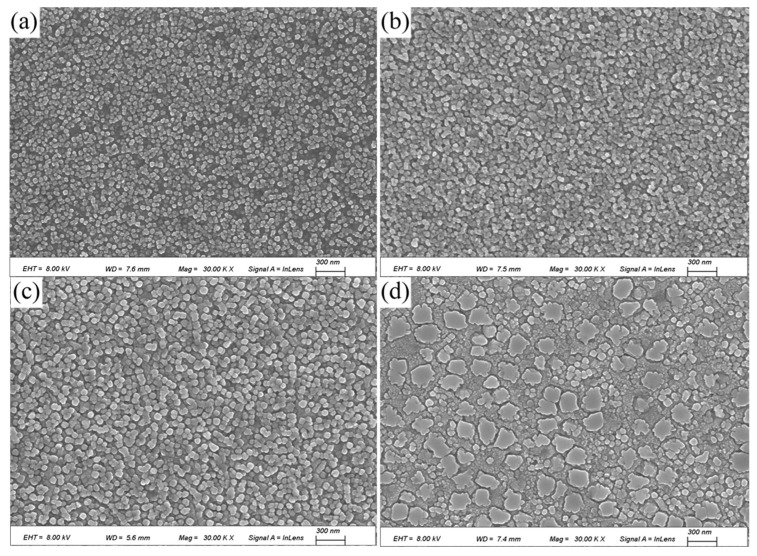
Direct aging for different times: (**a**) 4 h; (**b**) 8 h; (**c**) 12 h; (**d**) 16 h.

Both the Cu matrix and the coherent precipitates (Ni_3_Al) share a face-centered cubic (FCC) structure and exhibit very similar lattice parameters. Due to this similarity and the nanoscale size of the precipitates, therefore, SEM and TEM were used to observe and characterize the morphology of the precipitates. Figure 7 shows the SEM images of the alloy after direct aging at 723 K for 4 h, 8 h, 12 h, and 16 h. A large number of fine, high-density spherical precipitates are observed within the grains. These spherical precipitates are uniformly sized and dispersed. The size and morphology of the precipitates vary with aging time under different conditions.

**Figure 8:**
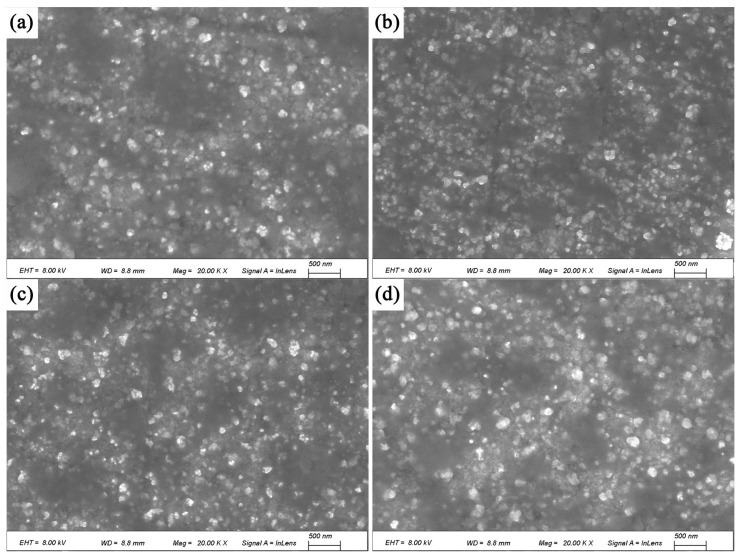
Solution aging for different times: (**a**) 4 h; (**b**) 8 h; (**c**) 12 h; (**d**) 16 h.

The SEM images of the alloy after solution treatment followed by aging for 4 h, 8 h, 12 h, and 16 h are shown in Figure 8. After aging for 4 h, strengthening precipitates begin to form, and their quantity increases with aging time. Compared with the direct aging, these precipitates are not uniformly dispersed, and their volume fraction is significantly lower. This is the primary reason why the mechanical properties and electrical conductivity of the solution-aged alloy are inferior to those of the directly aged alloy.

**Figure 9:**
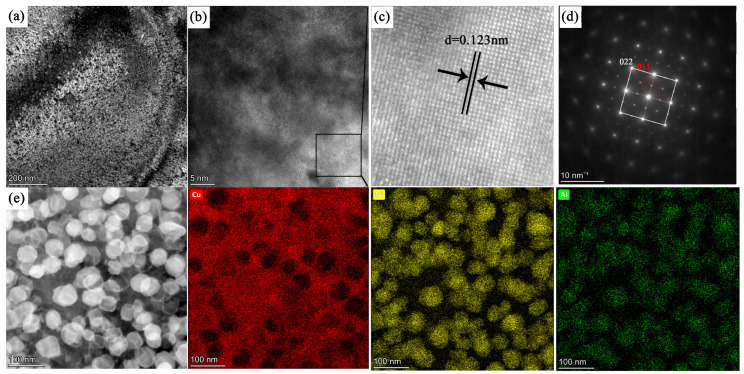
TEM images, (**a**) Bright field, (**b**) HRTEM, (**c**) Interplanar spacing for Ni_3_Al (**d**) selected area electron diffraction (SAED) pattern, and (**e**) EDS element mapping and point composition analysis of spherical morphology.

To further confirm the precipitation of the Ni_3_Al phase within the matrix, the microstructural features of the alloy were analyzed by transmission electron microscopy (TEM). Figure 9a is a transmission electron microscopy–dark field (TEM-DF) image; Figure 9b,c is HRTEM, which shows that interplanar spacing of the precipitate phase is 0.123 nm; and Figure 9d shows the selected area electron diffraction (SAED) pattern of the alloy in this aged condition. The superlattice diffraction spots corresponding to the precipitated phase can be clearly identified, thereby confirming the formation of Ni_3_Al during aging. The dispersed Ni_3_Al precipitates maintain excellent coherency with the matrix, and the crystallographic orientation relationship between the matrix and the ordered Ni_3_Al phase is as follows: (022)_matrix_//(011)_Ni3Al_, [001]_matrix_//[001]_Ni3Al_. Figure 9e presents the HAADF image and corresponding EDS elemental mapping of the alloy after direct aging at 723 K for 12 h. Numerous dispersed precipitate particles can be clearly observed to be uniformly distributed throughout the alloy matrix. These precipitates exhibit spherical-like morphology, with an average size of approximately 40 nm. According to the EDS analysis, the alloy consists of a copper-rich matrix and nickel–aluminum-rich precipitates.

## 4. Discussion

For precipitation-hardened alloys, traditional heat treatment methods typically involve solution treatment followed by aging to promote solute element precipitation. This study found that solution treatment leads to undesirable grain growth, which in turn reduces the alloy’s hardness and electrical conductivity.

This study leverages the unlimited miscibility of Cu-Ni alloys to induce spinodal decomposition during aging, resulting in the formation of periodic Ni-rich and Ni-depleted compositional modulations within the matrix. These compositional fluctuations facilitate the development of coherent nanoscale precipitates, thereby enhancing precipitation strengthening. After direct aging at 723 K, the alloy grains do not exhibit significant growth, and both hardness and electrical conductivity significantly increase. The superior mechanical properties observed in directly aged alloys are attributed to the combined effect of fine-grained structures and uniformly distributed precipitates. The former enhances strength via the Hall–Petch mechanism, while the latter ensures effective precipitation strengthening by providing a dense, coherent barrier to dislocation motion. Direct aging preserves the deformation-induced defect structure, which promotes both grain refinement and homogeneous nucleation of precipitates, resulting in a balanced improvement in strength and ductility.

The XRD patterns obtained after 15 min of direct aging at 723 K confirm the presence of spinodal decomposition. Due to the small wavelength of the microstructure formed by spinodal decomposition, it effectively strengthens by dispersion and impedes dislocation motion, thereby improving the alloy’s strength. Additionally, the precipitated phases formed by spinodal decomposition are coherent, preventing excessive dislocation pile-up, thus maintaining the material’s plasticity and toughness. The study also facilitated the transformation of the solute-rich and solute-depleted regions formed by spinodal decomposition into γ-Ni_3_Al phases.

Cu-15Ni-3Al alloy was aged for 15 min at 723 K, which indicates that spinodal decomposition occurred. Th spinodal wavelength (λ) was calculated using the following equation [12]:

$$\lambda =\frac{ha_{0}}{h^{2}+k^{2}+l^{2}}\cdot \frac{\tan\theta }{\Delta \theta }$$

where λ is the wavelength of the spinodal decomposition structure, which can be estimated using the Daniel–Lipson formula; *a*_0_ is the lattice parameter (3.615 nm); and (*hkl*) is the Miller index. In this study, based on (111), θ is the Bragg diffraction angle and Δθ is the distance between the diffraction peak side and the diffraction peak. Thus, the spinodal wavelength of the Cu-15Ni-3Al alloy is ~13.7 nm.

It is worth noting that in classical precipitation-hardened systems, the formation of γ-Ni_3_Al precipitates typically follows a nucleation-dominated sequence—beginning with primary γ′ precipitates at grain boundaries, followed by the intragranular formation of secondary and tertiary γ′ phases. However, in the Cu-15Ni-3Al alloy studied here, spinodal decomposition fundamentally alters this precipitation pathway. Rather than relying on heterogeneous nucleation at specific sites, spinodal decomposition occurs during aging and induces a continuous and spatially uniform compositional modulation within the matrix, resulting in the formation of periodic Ni-enriched and Ni-depleted regions. This promotes the homogeneous precipitation of coherent γ′ phases within the grains, significantly reducing the dominance of grain boundary precipitation and leading to a finer and more uniformly distributed microstructure. Therefore, spinodal decomposition not only enhances precipitation strengthening but also results in finer precipitate sizes and a more uniform and dispersed distribution of γ′ phases within the grains.

According to the different precipitation sequences of the γ phase, it is generally classified into three types: primary γ (γ_p_): coarse and irregularly shaped, located at the grain boundaries; secondary γ (γ_s_): mostly distributed inside the grains, with an octagonal shape, and during further decomposition, it divides into eight smaller parts; And Tertiary γ (γ_t_): fine and mainly located between large primary and secondary γ phases or in the gaps between secondary γ phases. In this study, during the early stages of aging, spherical γ phases precipitate inside the grains. As aging time increases, the spherical phases become uniformly sized and dispersed. After 16 h of aging, tertiary γ phases gradually grow into octagonal secondary γ phases, with tertiary γ phases distributed in the gaps between secondary γ phases [13,14,15].

## 5. Conclusions

Using Cu-15Ni-3Al alloy as the research object, the microstructural evolution during the aging process of the alloy was systematically studied. The aging precipitation behavior of the alloy was analyzed, and the effects of heat treatment conditions on the alloy’s aging precipitation behavior were discussed. The intrinsic relationship between the microstructure and the alloy’s mechanical properties was established, with the following conclusions drawn:

1. The hardness measurement of the forged alloy was 272.64 HV, and it reached the peak hardness of 316.3 HV after 12 h of aging. The optimal heat treatment process for this alloy was determined to be direct aging at 723 K for 12 h, which resulted in an alloy with excellent overall mechanical properties.
2. The directly aged samples exhibited significantly higher hardness compared to the solution-treated and aged counterparts. This enhancement is attributed to the combined effects of grain refinement and precipitation strengthening. As observed in the SEM and TEM images, the directly aged alloy retained a finer grain structure due to the absence of high-temperature solution treatment, which effectively suppressed grain growth. In addition, a denser distribution of nanoscale precipitates was qualitatively observed in the directly aged condition, contributing to a more effective barrier to dislocation motion. Therefore, the superior mechanical properties of the directly aged samples were the result of the synergistic contribution of both fine-grained microstructures and abundant, uniformly distributed precipitates.
3. After aging, spinodal decomposition occurred, and the solute-rich regions transformed into precipitates. The dispersed Ni_3_Al precipitates maintained a good coherent relationship with the matrix. The crystallographic orientation relationship between the matrix and the ordered Ni_3_Al phase was (022)_matrix_//(011)_Ni3Al_ and [001]_matrix_//[001]_Ni3Al_.

## Figures and Tables

**Table 1:** Nominal composition and mass of alloying elements used for Cu-15Ni-3Al alloy preparation.

Element	Ni	Al	Cu
Mass fraction (wt%)	15.0	3.0	Balance
Mass (kg)	4.5	0.9	24.6

**Table 2:** Heat treatment for the Cu-15Ni-3Al.

Heat Treatment Process	Aging Time	Heat Treatment Process	Aging Time
Direct aging in 723 K	2 h	Solution in 1173 K × 3 h and aging in 723 K	2 h
	4 h		4 h
	6 h		6 h
	…		…
	16 h		16 h

**Table 3:** Strength and elongation of the Cu-15Ni-3Al alloy in different states.

State	R_m_ (MPa)	R_p0.2_ (MPa)	Elongation (%)
Forging state	854	702	11.5
Direct aging 4 h	867	725	10.0
Direct aging 8 h	877	760	10.0
Direct aging 12 h	908	777	9.5
Direct aging 16 h	896	766	9.5

**Table 4:** Diffraction angles and lattice parameter of the Cu-15Ni-3Al alloy in different states.

	Diffraction Angles 2θ (°)	Lattice Parameter
Forging state	43.249	3.61899
Direct aging 4 h	43.263	3.61788
Direct aging 8 h	43.277	3.61676
Direct aging 12 h	43.289	3.61581

## Data Availability

The original contributions presented in this study are included in the article. Further inquiries can be directed to the corresponding author.

## References

[1] Mi X., Lou H., Xie H., Mo Y., Zhang W., Xiang C. (2023). Development Strategy for Advanced Copper-Based Materials in China. Chin. J. Eng. Sci..

[2] Jiang Y., Lou H., Xie H., Li T., Song K., Liu X., Yun X., Wang H., Xiao Z., Li Z. (2020). Development Status and Prospects of Advanced Copper Alloy. Chin. J. Eng. Sci..

[3] Weizong B., Longke B., Jie C., Li J., Xiang T., Yu B., Cai Z., Xie G. (2023). Heterogeneous metallic glass composites with a unique combination of strength, plasticity and conductivity. Int. J. Plast..

[4] Calum G.F., Katerina A., Emma M.H., Alison S.W., Nicholas G.J., Howard J.S. (2020). On the continuous and discontinuous precipitation of the L12 phase in Cu-Ni-Al alloys. Materialia.

[5] Li Z., Li X., Hu Y., Zheng Y., Yang M., Li N., Bi L., Liu R., Wang Q., Dong C. (2021). Cuboidal γ’ phase coherent precipitation-strengthened Cu–Ni–Al alloys with high softening temperature. Acta Mater..

[6] Semboshi S., Hariki R., Shuto T., Hyodo H., Kaneno Y., Masahashi N. (2021). Age-Induced Precipitating and Strengthening Behaviors in a Cu–Ni–Al Alloy. Metall. Mater. Trans. A.

[7] Dong H., Zhong R., Liu L., Wang Z., Yang C., Luo Z., Zhang W. (2023). The intergranular precipitation behavior of G phase in a high-performance complex cast Cu-Ni-Al alloy. Mater. Charact..

[8] Shen L., Li Z., Zhao Y., Wang Y., Dong Q., Wang M. (2016). Phase transformation behavior of Cu–10Ni–3Al–0.8Si alloy. Mater. Chem. Phys..

[9] Straumal B., Kilmametov A., López G., López-Ferreño I., Nó M.L., San Juan J., Hahn H., Baretzky B. (2019). High-pressure torsion driven phase transformations in Cu–Al–Ni shape memory alloys. Acta Mater..

[10] Lei Q., Li Z., Dai C., Wang J., Chen X., Xie J.M., Yang W.W., Chen D.L. (2019). Effect of aluminum on microstructure and property of Cu-Ni-Si alloys. Mater. Sci. Eng. A.

[11] Pereira C.E., Matlakhova A., Matlakhov A.N., de Araújo C.J., Shigue C.Y., Monteiro S.N. (2018). Reversible martensite transformations in thermal cycled polycrystalline Cu-13.7%Al-4.0%Ni alloy. J. Alloys Compd..

[12] Jiang K., Zhou Y., Yang R., Song K., Liu Y., Zhang Y., Yang S., Zhou F., Huang K., Liu D. (2023). Nanophase transformation and strengthening-toughening mechanism of the Cu–15Ni–8Sn alloy during aging. Mater. Res. Technol..

[13] Yao Z., Zhou B., Yao K., Wang H., Dong J., Davey T. (2020). Influence and sensitivity of temperature and microstructure on the fluctuation of creep properties in Ni-base superalloy. Materials.

[14] Hwang J.Y., Nag S., Singh A.R.P., Srinivasan R., Tiley J., Viswanathan G.B., Fraser H.L., Banerjee R. (2009). Compositional variations between different generations of γ′ precipitates forming during continuous cooling of a commercial nickel-base superalloy. Metall. Mater. Trans. A.

[15] Masoumia F., Jahazi M., Shahriari D., Cormier J. (2016). Coarsening and dissolution of γ′ precipitates during solution treatment of AD730™ Ni-based superalloy: Mechanisms and kinetics models. J. Alloys Compd..

